# Clinical outcomes one year after a digital musculoskeletal (MSK) program: an observational, longitudinal study with nonparticipant comparison group

**DOI:** 10.1186/s12891-022-05188-x

**Published:** 2022-03-11

**Authors:** Grace Wang, Manshu Yang, Mindy Hong, Jeffrey Krauss, Jeannie F. Bailey

**Affiliations:** 1 Hinge Health, Inc., 455 Market Street, Suite 700, CA 94105 San Francisco, USA; 2 Department of Psychology, University of Rhode Island, Chafee Hall 406, 142 Flagg Road, Kingston, RI 02881 USA; 3 Orthopaedic Surgery, University of California, San Francisco, 95 Kirkham St., San Francisco, CA 94122 USA

**Keywords:** Telemedicine, Musculoskeletal pain, Function, Depression, Anxiety

## Abstract

**Background:**

The evidence base for the impact of digital health on musculoskeletal (MSK) outcomes is growing, but it is unclear how much digital MSK programs address pain and function in the intermediate and long term.

**Methods:**

This observational study of digital MSK program participants versus nonparticipants (*n* = 2570) examined pain, function, depression, and anxiety at 3, 6, and 12 months, and health care use at 12 months. The intervention group engaged in a digital MSK program that included exercise, education, and coaching for at least 3 months. The nonparticipant group registered, but never started the program. We collected data in app or by emailed survey at 3, 6, and 12 months after registering for the program. We conducted descriptive analyses and unadjusted and adjusted regression modeling.

**Results:**

The odds ratio of achieving a minimally clinically important difference (MCID) in pain improvement for the intervention versus the nonparticipant group was 1.97 (95% CI: 1.28, 3.02; *p* = .002) at 3 months, 1.44 (95% CI: 0.91, 2.25; *p* = .11) at 6 months, and 2.06 (95% CI: 1.38, 3.08; *p* = .004) at 12 months in adjusted models. The odds ratio of achieving a MCID in functional improvement for the intervention versus the nonparticipant group was 1.56 (95% CI: 1.03, 2.38; *p* = .01) at 3 months, 1.55 (95% CI: 1.02, 2.37; *p* = .04) at 6 months, and 1.35 (95% CI: 0.89, 2.06, *p* = 0.16) at 12 months in adjusted models. For those with moderate to severe depression or anxiety at baseline, we observed statistically significant lower odds of moderate to severe depression or anxiety at 3 months, 6 months, and 12 months for the intervention versus the nonparticipant group in adjusted models (*p* < .05). At 12 months, the percentage with invasive, imaging, and conservative services was higher for the nonparticipant versus intervention group by 5.7, 8.1, and 16.7 percentage points, respectively (*p* < 0.05).

**Conclusions:**

A digital MSK program may offer participants sustained improvement in pain, depression, and anxiety with concomitant decreases in health care use.

**Supplementary Information:**

The online version contains supplementary material available at 10.1186/s12891-022-05188-x.

## Background

Chronic musculoskeletal (MSK) pain is a leading cause of disability and health care cost in the United States. Rates of osteoarthritis, back and neck pain, and other MSK disorders in the United States are among the highest in the world, with 134.5 million adults in the United States reporting MSK conditions in 2018 [1, 2].

Chronic MSK pain lasts or recurs for more than 3 months and may fluctuate in intensity over time [3]. Pain may be an aching and throbbing sensation in the background; or, pain may be intermittent, sharp, and stabbing [4]. Chronic MSK pain may hinder activities of daily living, including walking, getting up from sitting, opening a jar, or reaching overhead. Furthermore, chronic MSK pain often occurs with and exacerbates depression and anxiety [5]. Depression and anxiety can also influence pain severity and duration [6–9].

To improve MSK function, and reduce pain and associated comorbidities, evidence-based clinical guidelines typically recommend conservative therapies before invasive treatments [10]. First line, conservative therapies include exercise and education because of their safety and impact on outcomes [4, 11–14]. For example, a meta-analysis of 3514 trial participants found that exercise reduced lower back pain an average of 10.7 points out of 100 and reduced functional limitations by 10.2 points out of 100 versus control groups [11]. Studies have also demonstrated the effectiveness of pain neuroscience education with exercise on significantly decreasing pain, disability, kinesiophobia, and pain catastrophizing among persons with chronic MSK pain [13].

Digital health approaches are now used to deliver conservative therapies via interactive tools. These approaches may help to facilitate care access because of the convenience of digital health (e.g., members can access services at all hours and locations and during periods with severe pain symptoms) [15]. In addition, participants of digital health programs have seen significant improvements in knee and back pain [16, 17]. For example, Du et al’s systematic review found moderate-quality evidence that digital MSK programs resulted in statistically significant back pain improvements at immediate and short-term follow-ups and functional improvement at immediate follow-ups when compared to waiting-list, usual care, or active controls (e.g., health education) [17].

Although the evidence base for the impact of digital health on MSK outcomes is growing, previous research is limited in the following ways. It is still unclear how much digital MSK programs address pain and function in the intermediate and long term. The impact on depression and anxiety is not yet well established. Many previous digital MSK program evaluations are small randomized controlled trials with high internal validity, but questions remain about how engagement in real world settings affects program outcomes. Finally, researchers have not examined how participation in digital MSK programs influences use of traditional, in-person health care services.

To address these gaps, our study focused on three objectives. The primary objective was to examine pain improvement at 3, 6, and 12 months for digital MSK program participants versus nonparticipants. The secondary objective was to examine functional and mental health outcomes at 3, 6, and 12 months for digital MSK program participants versus nonparticipants. For both these objectives, we hypothesized that digital MSK program participants would have better outcomes versus nonparticipants at 3, 6, and 12 months. Finally, we explored self-reported health care use for digital MSK program participants versus nonparticipants at 12 months. Results from this study provide evidence about whether a digital MSK program offers participants sustained improvement in pain, function, and mental health with concomitant decreases in health care use.

## Methods

### Study design

We conducted an observational, longitudinal cohort study design comparing digital MSK program participants versus nonparticipants.

### Digital MSK program description

Employers offered the digital MSK program to employees and dependents as a health or wellness benefit. Recruitment was conducted through email, workplace posters or presentations, and mailings. Registration involved creating a member profile and completing a baseline application online. After registering, participants had access to the program for 1 year. They could renew after 1 year if their employer continued to offer the program as a health benefit.

The digital MSK program’s goal was to help participants manage chronic MSK pain by offering exercise therapy, education, and personal health coaching. Materials provided to registrants included tablet computers with a program app and wearable motion sensors (InvenSense MPU-6050, TDK Electronics, Tokyo, Japan).

The program delivered exercise therapy and education through “playlists” accessed in the app. Each playlist presented three to five exercises that were specific to back, knee, shoulder, hip, or neck pain. The curriculum included more than 60 distinct stretching, strengthening, balance and mobility exercises. Each playlist included stretching, strengthening, balance and mobility activities. The playlist presented 1 to 2 sets of 3 to 10 repetitions depending on the difficulty and type of exercise, and we recommended completing playlists at least 3 times per week. Animations and videos within the app demonstrated how to perform exercises, the number of repetitions, and how long to hold positions. By pairing with the sensors, the app displayed body position during exercises in real-time and provided feedback about the appropriate range of movement. As participants progressed through the program, the playlists presented more challenging exercises and/or added more repetitions. Progression was individualized based on how often a member engaged and completed an exercise playlist. For example, the app introduced new playlists after the member completed an earlier playlist three times.

After the exercises, the playlist delivered educational resources about MSK pain-related topics, such as pain neuroscience, movement, treatment options, coping, lifestyle changes, relaxation, social support, and habit creation. Each playlist was designed to take less than 15 min, and health coaches (described next) actively encouraged participants to complete at least three playlists per week for the first 3 months. Our program retains between 67 to 83% of members through month 3 depending on age group, with members of different age groups averaging 26 to 45 exercise sessions through month 3 [18]. Participants then had access to the program for the remainder of the year with decreased coaching. As a wholly virtual program, participants could choose when and where to complete playlists.

In addition to exercise and education, the digital MSK program provided personal support to adhere to the program. Each participant was matched to a personal, certified health coach. Coaches initiated contact with participants via text message and communicated with members asynchronously over time via text message, email, or in-app messaging. In addition, participants could schedule up to three phone calls with health coaches. The coach acted as a supportive accountability partner to help participants build an exercise habit. Coaches worked with participants to set goals, identify challenges to performing exercises, and implement strategies to overcome challenges. Coaches also answered questions about the technology, playlists, and educational resources. Coaches provided support for the duration of a participant’s engagement with the program. The intervention group members in the sample sent a total of 88,565 messages to coaches by month 3, averaging 22 messages per person. This is consistent with our previous reports [18]. Members could also take part in virtual discussion forums with 20 to 30 others.

### Study participants

Study participants met the following criteria: created an account; provided informed research consent; age 18 or older; pain in the low back, knee, shoulder, hip, or neck; baseline visual analog scale (VAS) pain score greater than 0; pain lasted for at least 12 weeks; and member covered by employer’s health plan. Exclusion criteria were signs of fracture, joint instability, infection, cancer, and cauda equina syndrome. We used the information provided in the baseline application to determine whether participants met these criteria. We did not require formal diagnoses from medical providers.

At the time of program registration, we provided an information sheet about the program and our research. Only participants who acknowledged reviewing the information sheet and agreed to the research provisions were included in this study. The study (reference number #20160949) was reviewed and approved by WIRB-Copernicus Group® Institutional Review Board (OHRP/FDA IRB registration number IRB00000533) at WIRB-Copernicus Group® (1019 39th Avenue SE Suite 120, Puyallup, Washington 98,374–2115). Study subjects acknowledged online that they provided informed consent before study inclusion. The ethics committee approved the waiver of written documentation of informed consent because the program is entirely digital.

This study was designed to include multiple follow-up time points with all final data collection occurring in quarter (Q) 2–2021. Thus, we retrospectively identified three separate cohorts. Cohort 1 registered in Q2–2020, Cohort 2 registered in Q4–2020, and Cohort 3 registered in Q1–2021. Within each of these cohorts, nonparticipants registered for the program but did not complete any exercise therapy sessions and did not access any educational articles. The intervention group completed exercise therapy sessions or accessed educational articles through month 3 (completer subgroup) or completed exercise therapy sessions or accessed educational articles in months 3 to 6 (long term subgroup).

To sample, we stratified on body region (back, knee, shoulder, hip, neck), cohort (cohort 1, cohort 2, cohort 3), and group (nonparticipants, completer, long term). Then we randomly sampled *n* = 114 per region-cohort-group. After excluding individuals who did not provide informed consent, Cohorts 1 included n = *n* = 570 nonparticipants and *n* = 1140 digital MSK program participants. Cohort 2 included n = *n* = 535 nonparticipants and *n* = 1057 digital MSK program participants. Cohort 3 included *n* = 545 nonparticipants and *n* = 523 digital MSK program participants.

Table 1 shows each cohort’s progression from registration through final data collection. For example, Cohort 1 registered for the program in Q2–2020 and completed 3, 6, and 12 month data collection in app in Q3–2020, Q4–2020, and Q2–2021, respectively. In Q2–2021, we also emailed surveys to all nonparticipants and intervention group members who did not enter 12-month follow-up data in app.

**Table 1:** Cohort activities over time

	Q2_2020	Q3_2020	Q4_2020	Q1_2021	Q2_2021
Cohort 1	Registers; intervention group completes program	Completes 3 month follow-up in app	Completes 6 month follow-up in app		Completes 12 month follow-up in app or email survey
Cohort 2			Registers; intervention group completes program	Completes 3 month follow-up in app	Completes 6 month follow-up in app or email survey
Cohort 3				Registers; intervention group completes program	Completes 3 month follow-up in app or email survey

### Variables

The following section describes outcomes, exposures, and covariates.

#### Outcomes

The primary outcome was achieving a minimally clinical important difference (MCID) in pain improvement (no/yes). To create this dichotomous variable, we gathered baseline and follow-up responses to the question “Over the past 24 hours, how bad was your [back/knee/shoulder/hip/neck] pain?” from 0 (none) to 100 (worst imaginable). Next, we calculated the change from baseline to follow-up. A person achieved MCID in pain improvement if they showed at least a 20 point decrease or 30% improvement [19].

We included three secondary outcomes. One secondary outcome was achieving a MCID in functional improvement (no/yes). To create this dichotomous variable, we gathered baseline and follow-up responses to the 11-item Roland Morris Disability Questionnaire (RMDQ-11, back only), Knee injury and Osteoarthritis Outcome Score Physical Function Short form (KOOS-PS, knee only), Hip Disability and Osteoarthritis Outcome Score Physical Function Short form (HOOS-PS, hip only), Shoulder Pain and Disability Index (SPADI, shoulder only), Neck Pain and Disability Scale short form (sf-NPAD, neck only). Next, we calculated the change from baseline to follow-up. A person achieved MCID in functional improvement if they showed at least: 30% improvement on the RMDQ-11 [20, 21]; or 8 point improvement on the KOOS-PS [22–24]; or 9.3 point improvement on the HOOS-PS [25, 26]; or 13 point improvement on the SPADI [27–29]; or 12 point improvement on the sf-NPAD [30, 31]; or no limitations at follow-up.

Another secondary outcome was moderate or severe depression at follow-up (no/yes). To create this dichotomous variable, we first gathered baseline and follow-up responses to the Patient Health Questionnaire 2-item scale (PHQ-2). Those who screened positive (i.e., score of 3 or higher) on the PHQ-2 received the PHQ 8-item scale (PHQ-8). Moderate or severe depression was a score of 10 or higher on the PHQ-8. The last secondary outcome was moderate or severe anxiety (no/yes). To create this dichotomous variable, we first gathered baseline and follow-up responses to the Generalized Anxiety Disorder 2-item scale (GAD-2). Those who screened positive (i.e., score of 3 or higher) on the GAD-2 received the GAD 7-item scale (GAD-7). Moderate or severe anxiety was a score of 10 or higher on the GAD-7. Cutoffs at 10 points have been shown to have acceptable performance for identifying anxiety and depression [32–34].

We explored health care utilization among emailed survey respondents at 12 months. We asked: Since signing up for [the digital MSK program] about 12 months ago, have you had any of the following for your <back/knee/shoulder/hip/neck> pain? Respondents indicated whether or not they had any of the following services: conservative care (e.g., office visit with a doctor or a physical therapist), invasive procedures (e.g., emergency department or urgent care center visit, overnight stay in a hospital, injections, or surgery), or imaging (e.g., MRI, scan, X-ray).

#### Exposures

The nonparticipant group registered, but did not complete exercise therapy sessions and did not access educational articles. The intervention group completed exercise therapy sessions or accessed educational articles through month 3 (completer subgroup) or completed exercise therapy sessions or accessed educational articles in months 3 to 6 (long term subgroup). Exercise completion and educational article access were recorded when participants used the app. Therefore, we did not record information about exercises completed outside the app.

#### Confounders

Model covariates included cohort, gender, age, exercise frequency per week at baseline (less than 1 h, 1 to 2.5 h, more than 2.5 h), BMI at baseline, pain region (back, knee, shoulder, hip, neck), baseline anxiety, baseline depression, and state of residence.

### Data sources

Baseline data were collected via an online survey that the nonparticipant and intervention groups completed at program registration.

The intervention group took part in the digital MSK program and entered data in app at follow-up time points. If intervention group members did not enter data in app, trained data collectors from a data collection firm representing the digital MSK program emailed the intervention group members surveys about current pain, function, and mental health status at the final follow-up time point for their cohort (e.g., 12 months for cohort 1). For example, cohort 1 may have entered data in app at 3 months, in app at 6 months, and then by emailed survey at 12 months. The data collection firm emailed and called nonresponders with reminders to complete the emailed survey. Intervention group members also had the option to complete the survey by phone.

The data collection firm also emailed the nonparticipant group surveys about their current pain, function, and mental health status at the final follow-up time point for their cohort. That is, the cohort 1 nonparticipants completed emailed surveys at 12 months, cohort 2 nonparticipants completed emailed surveys at 6 months, and cohort 3 nonparticipants completed emailed surveys at 3 months. The data collection firm emailed and called nonresponders with reminders to complete the emailed survey, and the nonparticipant group could choose to complete the survey by phone. Upon completion of emailed surveys, nonparticipant and intervention group members received $25 gift cards.

### Study size

We estimated a sample size that enabled pairwise comparisons among the groups at each follow-up time point. The minimally clinically important difference for VAS pain is 20 points on a scale of 0 to 100 [19]. Based on previous results from RCTs, we assumed a standard deviation of 22 for the VAS scores within each group for power calculation [35]. Bonferroni correction was used to account for multiple comparisons among groups. To achieve 80% statistical power, we needed at least 47 participants in each group to detect a 15-point difference in VAS (Cohen’s d = 0.68), given an overall Type I error rate of 0.05.

### Statistical methods

Summary statistics were estimated for gender, age, exercise frequency per week at baseline, BMI at baseline, baseline anxiety, and baseline depression. Descriptive statistics were reported at 3, 6 and 12 months for the percentage of patients who achieved a MCID in pain improvement, a MCID in functional improvement, moderate to severe depression, and moderate to severe anxiety. For these dichotomous outcomes, we used a two-proportions z-test to compare the intervention group versus the nonparticipant group at each timepoint.

We conducted unadjusted and adjusted regression analyses. For the primary outcome of achieving MCID in pain, we conducted multivariable-adjusted logistic regression at 3, 6 and 12 months controlling for gender, age, state of residence, exercise frequency per week at baseline (less than 1 h, 1 to 2.5 h, more than 2.5 h), pain region (back, knee, shoulder, hip, neck), cohort, baseline BMI, baseline anxiety, and baseline depression.

For the secondary outcome of achieving MCID in function, multivariable-adjusted logistic regression at 3, 6 and 12 months controlled for gender, age, state of residence, exercise frequency per week at baseline, cohort, baseline pain, baseline BMI, baseline anxiety, and baseline depression. We examined moderate to severe depression or anxiety at follow-up among the subset with moderate to severe depression/anxiety at baseline. The multivariable-adjusted logistic regression models at 3, 6 and 12 months controlled for gender, age, state of residence, exercise frequency per week at baseline, baseline pain, and baseline BMI.

We conducted a subgroup analysis examining descriptive statistics for program completers versus long term engagers. For dichotomous MCID in pain improvement, two-proportions z-tests were used to compare the program completers versus the long term users group at 3, 6 and 12 months.

The primary analysis employed complete case analysis, i.e., excluded missing values. To address missing data, sensitivity analysis for MCID in pain improvement was performed using multiple imputation by chained equations (*n* = 10 imputations) assuming data were missing at random. All variables from the regression model were included in the imputation model.

All analyses were performed in R version 4.0.5 (R Core Team, Vienna, Austria).

## Results

### Flowchart

**Fig. 1:**
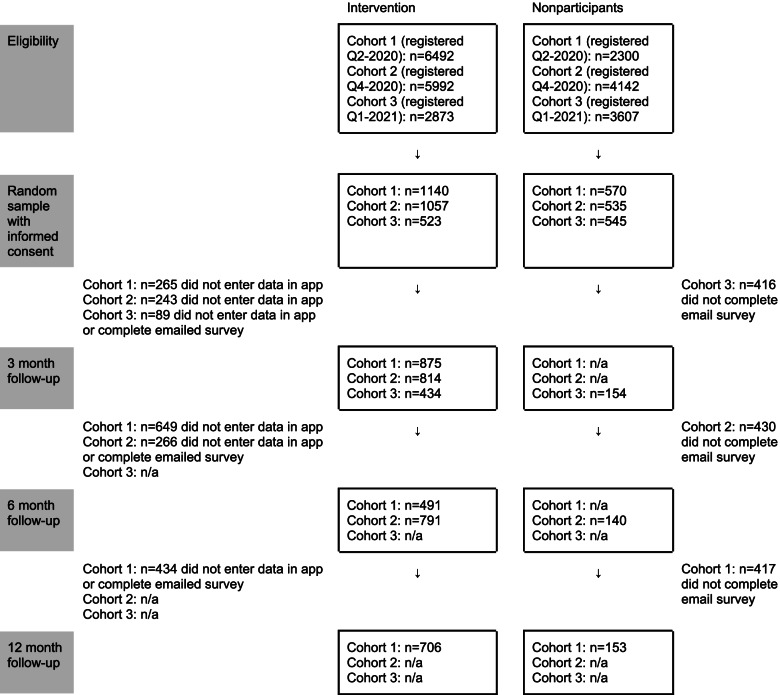
Flowchart, by cohort

Figure 1 reports on the number of intervention group members and nonparticipants at each study stage, by cohort. Overall, we achieved a 70% response rate at 3 months (2277/3265), a 52% response rate at 6 months (1422/2732), and a 50% response rate at 12 months (859/1710).

### Sample characteristics

Table 2 shows the characteristics of sampled nonparticipant and intervention groups at baseline. About 63% of the intervention group is female versus 59% in the nonparticipant group. The intervention group has a mean age of 49.3 years old versus 45.8 years in the nonparticipant group. Over 70% of the intervention group was overweight or obese compared to 79% of the nonparticipant group. Compared to the intervention group, a larger percentage of the nonparticipant group exercised less than 1 h per week, experienced moderate to severe anxiety, and experienced moderate to severe depression. Characteristics of the analytic sample who responded to follow-up surveys are similar to the study sample (Additional File 1).

**Table 2:** Study sample characteristics at baseline

	Comparison (***N*** = 1650)	Intervention (***N*** = 2720)	Total (***N*** = 4370)
**Gender**			
Female	975 (59.1%)	1704 (62.7%)	2679 (61.3%)
Male	662 (40.1%)	995 (36.6%)	1657 (37.9%)
Other	3 (0.2%)	6 (0.2%)	9 (0.2%)
Prefer Not to Answer	10 (0.6%)	15 (0.6%)	25 (0.6%)
Age*			
Mean (SD)	45.8 (12.4)	49.3 (12.1)	48.0 (12.3)
Median [Min, Max]	45.8 [18.3, 87.5]	50.4 [18.1, 86.2]	48.8 [18.1, 87.5]
BMI*			
Underweight (< 18.5)	16 (1.0%)	36 (1.3%)	52 (1.2%)
Normal (18.5–24.9)	326 (19.8%)	772 (28.4%)	1098 (25.1%)
Overweight (25.0–29.9)	502 (30.4%)	865 (31.8%)	1367 (31.3%)
Obese (> 30.0)	806 (48.8%)	1047 (38.5%)	1853 (42.4%)
Exercise Frequency*			
Less than 1 h	630 (38.2%)	729 (26.8%)	1359 (31.1%)
1 to 2.5 h	647 (39.2%)	1158 (42.6%)	1805 (41.3%)
More than 2.5 h	373 (22.6%)	833 (30.6%)	1206 (27.6%)
Percent with Moderate/Severe Anxiety*	421 (25.5%)	461 (17.0%)	882 (20.2%)
Percent with Moderate/Severe Depression*	291 (17.6%)	282 (10.4%)	573 (13.1%)

* *p* < 0.05 comparing groups for chi-square test of independence for categorical variables and two-sample t-test for continuous variables

### Descriptive results

**Fig. 2:**
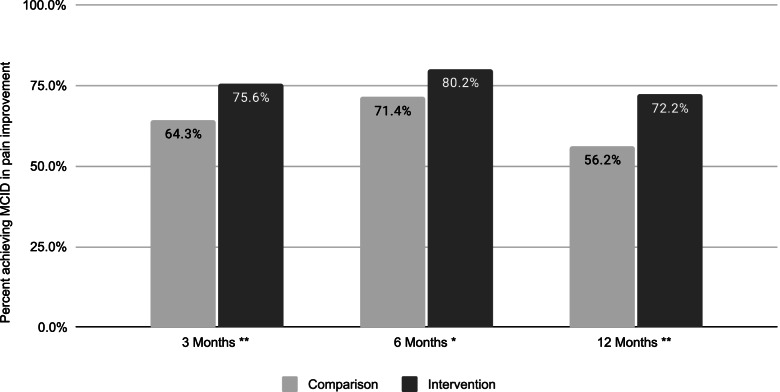
Percent achieving MCID in pain, by follow-up time point. Nonparticipant group denominator was *n* = 154 at 3 months, *n* = 140 at 6 months, and *n* = 153 at 12 months. Intervention group denominator was *n* = 2123 at 3 months, *n* = 1282 at 6 months, and *n* = 706 at 12 months. Differences between comparison and intervention group were statistically significant at * *p* < =.01 and ** *p* < =.001

The percentage achieving MCID in pain improvement was significantly higher for the intervention group versus the nonparticipant group by 11.3 percentage points at 3 months, 8.8 percentage points at 6 months, and 16.0 percentage points at 12 months (*p* < .05, Fig. 2). In addition, pain scores decreased from 48.7 points (SD 22.7) at baseline to 24.4 (SD 25.5) at 3 months, 26.3 (SD 26.3) at 6 months, and 32.7 (SD 28.9) at 12 months among the nonparticipant group (data not shown). The intervention group’s pain decreased from 45.0 (SD 22.0) at baseline to 18.2 (SD 19.6) at 3 months, 14.3 (SD 17.8) at 6 months, and 22.1 (SD 23.3) at 12 months (data not shown).

**Fig. 3:**
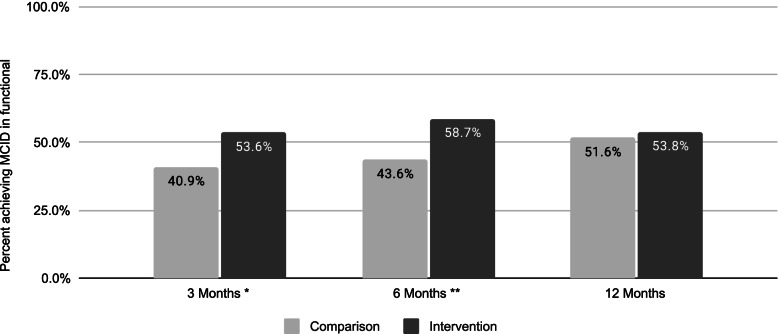
Percent achieving MCID in function, by follow-up time point. Nonparticipant group denominator was *n* = 154 at 3 months, *n* = 140 at 6 months, and *n* = 153 at 12 months. Intervention group denominator was *n* = 2123 at 3 months, *n* = 1282 at 6 months, and *n* = 706 at 12 months. Differences between comparison and intervention group were statistically significant at * *p* < =.01 and ** *p* < =.001

On secondary outcomes, the percentage achieving a MCID in functional improvement was higher for the intervention group versus the nonparticipant group by 12.6 percentage points at 3 months, 15.2 percentage points at 6 months, and 2.2 percentage points at 12 months (Fig. 3).

**Fig. 4:**
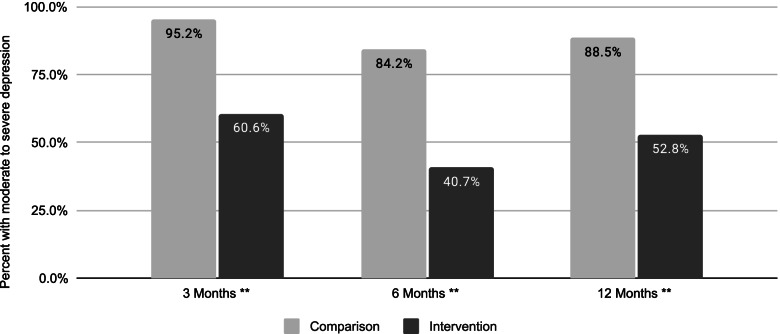
Percent with moderate to severe depression, among those with moderate to severe depression at baseline, by follow-up time point. Nonparticipant group denominator was *n* = 21 at 3 months, *n* = 19 at 6 months, and *n* = 26 at 12 months. Intervention group denominator was *n* = 175 at 3 months, *n* = 118 at 6 months, and *n* = 89 at 12 months. Differences between comparison and intervention group were statistically significant at * *p* < =.01 and ** *p* < =.001

Among those with moderate to severe depression at baseline, the percentage with moderate to severe depression at followup was significantly higher for the nonparticipant group versus the intervention group by 34.7 percentage points at 3 months, 43.5 percentage points at 6 months, and 35.7 percentage points at 12 months (*p* < =0.001, Fig. 4).

**Fig. 5:**
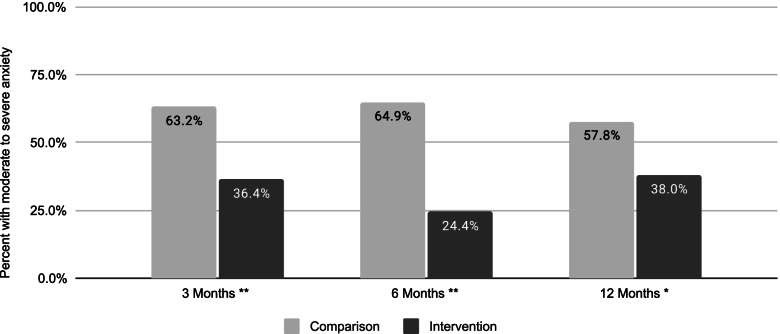
Percent with moderate to severe anxiety, among those with moderate to severe anxiety at baseline, by follow-up time point. Nonparticipant group denominator was *n* = 38 at 3 months, *n* = 37 at 6 months, and *n* = 45 at 12 months. Intervention group denominator was *n* = 297 at 3 months, *n* = 205 at 6 months, and *n* = 166 at 12 months. Differences between comparison and intervention group were statistically significant at * *p* < =.01 and ** *p* < =.001

Among those with moderate to severe anxiety at baseline, the percentage with moderate to severe anxiety at followup was significantly higher for the nonparticipant group versus the intervention group by 26.8 percentage points at 3 months, 40.5 percentage points at 6 months, and 19.8 percentage points at 12 months (*p* < =0.01, Fig. 5).

**Fig. 6:**
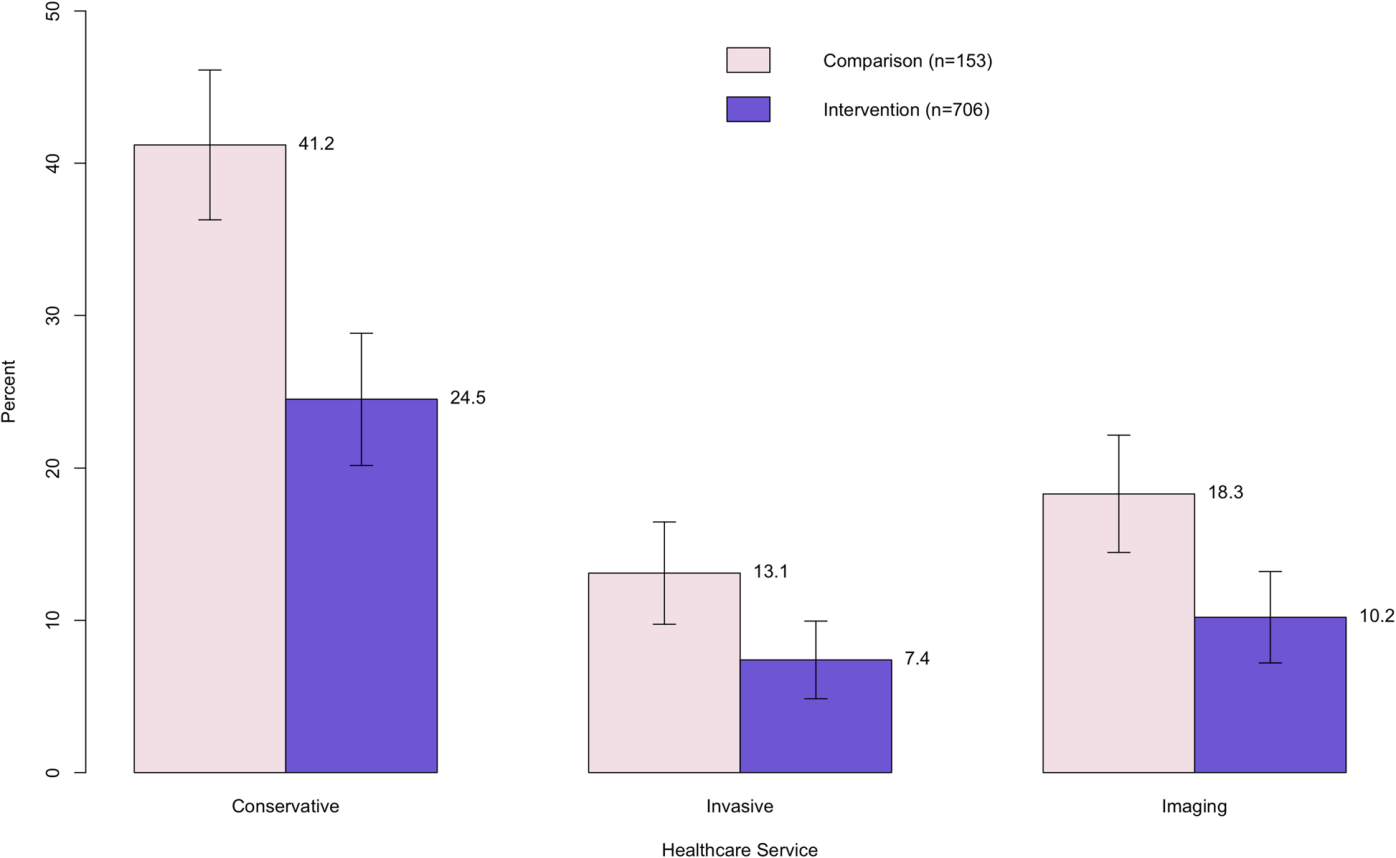
Descriptive results for self reported health care use at 12 months

Figure 6 shows descriptive results for self-reported health care use. At 12 months, the percentage with conservative (e.g., office or therapy visit), invasive (e.g., surgery, injections, emergency room), and imaging services was higher for the nonparticipant group versus the intervention group by 16.7 percentage points, 5.7 percentage points, and 8.1 percentage points, respectively (*p* < 0.05).

### Main results

Table 3 shows results from unadjusted and adjusted models for primary and secondary outcomes. In adjusted models, we observed higher odds of achieving a MCID in pain improvement at 3 months (OR: 1.97; 95% CI: 1.28, 3.02; *p* = .002), at 6 months (OR: 1.44; 95% CI: 0.91, 2.25; *p* = .11), and 12 months (OR: 2.06; 95% CI: 1.38, 3.08; *p* = .004) for the intervention versus the nonparticipant group.

**Table 3:** 3, 6, and 12 month results in unadjusted and adjusted models comparing the intervention group to the nonparticipant group (reference)

Primary Outcome	Timepoint	Unadjusted OR (95% CI)	Adjusted OR (95% CI)	***p***-value
Pain (MCID)	3 months	1.72 (1.21, 2.42)	1.97 (1.28, 3.02)	0.002
	6 months	1.62 (1.08, 2.38)	1.44 (0.91, 2.25)	0.113
	12 months	1.97 (1.37, 2.82)	2.06 (1.38, 3.08)	0.004
**Secondary Outcome**				
Function (MCID)	3 months	1.67 (1.20, 2.33)	1.56 (1.03, 2.38)	0.010
	6 months	1.84 (1.30, 2.63)	1.55 (1.02, 2.37)	0.041
	12 months	1.14 (0.80, 1.61)	1.35 (0.89, 2.06)	0.160
Moderate or severe depression	3 months	0.41 (0.25, 1.43)	0.27 (0.12, 0.60)	0.002
	6 months	0.33 (0.19, 0.62)	0.41 (0.18, 0.91)	0.026
	12 months	0.40 (0.24, 0.70)	0.35 (0.19, 0.65)	0.001
Moderate or severe anxiety	3 months	0.34 (0.21, 1.80)	0.21 (0.09, 0.43)	< 0.001
	6 months	0.22 (0.13, 0.37)	0.15 (0.07, 0.31)	< 0.001
	12 months	0.48 (0.29, 0.80)	0.34 (0.19, 0.61)	< 0.001

In adjusted models, we observed higher odds of achieving MCID in functional improvement at 3 months (OR: 1.56; 95% CI: 1.03, 2.38; *p* = .01), 6 months (OR: 1.55; 95% CI: 1.02, 2.37; *p* = .04), and 12 months (OR: 1.35; 95% CI: 0.89, 2.06; *p* = 0.16) for the intervention versus the nonparticipant group.

For the subgroup with moderate or severe depression at baseline, we observed lower odds of moderate or severe depression at 3 months (OR: 0.27; 95% CI: 0.12, 0.60; *p* = .002), 6 months (OR: 0.41; 95% CI: 0.18, 0.91; *p* = 0.026), and 12 months (OR: 0.35; 95% CI: 0.19, 0.65; *p* = 0.001) for the intervention versus the nonparticipant group in adjusted models. For the subgroup with moderate or severe anxiety at baseline, we observed lower odds of moderate or severe anxiety at 3 months (OR: 0.21; 95% CI: 0.09, 0.43; *p* < .001), 6 months (OR: 0.15; 95% CI: 0.07, 0.31; *p* < .001), and 12 months (OR: 0.34; 95% CI: 0.19, 0.61; *p* < .001) for the intervention versus the nonparticipant group in adjusted models.

### Subgroup analyses

We examined intervention subgroups defined by engagement duration. The completer subgroup was defined as those completing exercise sessions only through month 3, while the long term subgroup also completed exercise sessions in months 4 through 6. We did not detect significantly different percentages in the two subgroups achieving a MCID in pain improvement at 3 months. But, the percentage achieving a MCID in pain improvement was higher for the long term group versus the completer group by 10 percentage points at 6 months, and 9 percentage points at 12 months (*p* < =.004) (Additional File 2).

### Sensitivity analyses

We conducted a sensitivity analysis applying a multiple imputation by chained equations approach for MCID in pain improvement. The odds ratios for the intervention versus the nonparticipant group was 1.37 at 3 months (95% CI: 1.08, 1.73; *p* = 0.008), 0.96 at 6 months (95% CI:0.78, 1.19; *p* = 0.12), and 1.38 at 12 months (95% CI: 1.12, 1.69; *p* = .002) in adjusted models.

## Discussion

This observational study examined pain, function, depression, and anxiety at 3, 6, and 12 months and health care use during the 12 months after starting a digital MSK program versus a nonparticipant group. We found significant associations between the intervention and clinically meaningful pain improvement at 3 and 12 months and functional improvement at 3 and 6 months. Among the subset of persons with moderate or severe depression or anxiety at baseline, the intervention group was significantly associated with symptom improvement at all timepoints. Finally, a smaller percent of intervention group members used invasive, imaging, or conservative services at 12 months versus the nonparticipant group.

Participation in a digital MSK program was significantly associated with a MCID in pain improvement in the short and long term. Based on adjusted models, the percentage that achieved a MCID in pain improvement was higher for the intervention versus the nonparticipant group by 14 percentage points at 3 months, 14 percentage points at 6 months, and 12 percentage points at 12 months. Over half of the intervention and nonparticipant groups experienced meaningful pain improvements over time. We propose that people with chronic pain likely registered for the digital MSK program while experiencing elevated pain. By 12 months, the nonparticipant group achieved pain improvement with the help of traditional health care services, but still did not experience the same results as the intervention group.

Our pain improvement findings were consistent with previous research about the effectiveness of exercise training on decreasing chronic MSK pain [36–40]. For low back pain, Quentin et al’s meta analysis of 13 studies reported that home-based exercise training decreased low back pain versus control groups (effect size = − 0.97, 95% CI − 1.14 to − 0.79) [36]. Skelly et al’s meta analysis reported that exercise was associated with decreased back pain versus control groups at short-term (11 trials, pooled difference − 1.21 on a 0 to 10 scale, 95% CI − 1.77 to − 0.65), intermediate-term (5 trials, − 0.85, 95% CI − 1.67 to − 0.07), and long-term (1 trial, difference − 1.55, 95% CI − 2.76 to − 0.34). For knee osteoarthritis, the same report showed pain improvement for intervention versus control groups in the short term (8 trials, − 0.47, 95% CI − 0.86 to − 0.10); intermediate term (11 trials, − 1.34, 95% CI − 2.12 to − 0.54), and long term (4 trials, − 0.30, 95% CI − 0.49 to 0.00). For hip osteoarthritis, exercise showed a small improvement in only short-term pain compared with usual care (3 trials, − 0.30, 95% CI − 0.70 to − 0.02) [40]. Narrative systematic reviews have also found that exercise is associated with pain improvement for hip osteoarthritis, subacromial shoulder pain, and chronic pain from multiple diagnoses [37–39].

Participation in a digital MSK program was significantly associated with functional improvement in the short and medium term. A significantly larger percentage of digital MSK program participants showed meaningful functional improvement versus a nonparticipant group at 3 and 6 months. Other studies have examined the benefits of multidisciplinary and exercise programs on function for people with chronic pain conditions [39–41]. A review of Cochrane reviews found that function was significantly improved for persons with chronic pain after exercise interventions in 14 reviews (small to moderate effect sizes) [39]. For low back pain, Skelly et al’s meta analysis found that exercise showed improvement in only short-term function compared with control groups (10 trials, pooled standardized mean difference (SMD) − 0.31, 95% CI − 0.50 to − 0.13). For knee osteoarthritis, exercise was associated with improved function compared with control groups in the short term (8 trials, pooled SMD − 0.29, 95% CI − 0.46 to − 0.11), intermediate term (11 trials, pooled SMD − 0.63, 95% CI − 1.17 to − 0.10), and long term (4 trials, pooled SMD − 0.22, 95% CI − 0.34 to − 0.08). In hip osteoarthritis, exercise was associated with functional improvement versus control groups in the short term (3 trials, pooled SMD − 0.33, 95% CI − 0.58 to − 0.11), intermediate term (2 trials, pooled SMD − 0.28, 95% CI − 0.55 to 0.02), and long term (1 trial, SMD − 0.37, 95% CI − 0.74 to − 0.01) [40]. However, in our study, the odds ratios for MCID in function for the intervention versus the nonparticipant group declined over time. One reason may be related to ongoing participation in the digital MSK program. To sustain functional improvement, we hypothesize that participants may need to regularly complete exercise sessions over time. To continue to show improvements over time, the digital MSK program may need to include more motor skill training in addition to strength and flexibility exercises. For example, van Dillen et al. successfully used motor skill training to target how people performed functional activities, and this approach resulted in functional improvements that endured [42].

Participation in a digital MSK program was significantly associated with improvements in depression and anxiety at all timepoints among persons with moderate to severe depression and anxiety at baseline. In contrast to our findings, previous research estimates about exercise and depression have been unclear [43]. One reason may be that exercise may be less effective for people with depression in the absence of chronic MSK pain. A digital MSK program may address MSK pain through exercise and engagement and thus reduce depressive symptoms exacerbated by MSK pain. Our study’s results on anxiety were consistent with research showing the positive and lasting effect of exercise on anxiety [44]. The program’s low intensity exercises and educational articles may have helped participants to experience less fear of movement, more self-efficacy about managing pain, and “time out” away from anxious thoughts.

Significantly fewer participants of a digital MSK program reported health care use at 12 months versus a nonparticipant group. One possible reason for this result was that a digital MSK program prevented the need for health care services, especially invasive services, because of improved pain and function outcomes over time. A digital MSK program may have also acted as a substitute for usual care, especially conservative care. That is, participants may have practiced the exercises and stretches through the program instead of going to in-person therapy. Because these cohorts registered for the program during the COVID-19 pandemic, the percent using in-person health care was likely lower than usual in both groups, but it is unclear whether the pandemic influenced health care use differently in the nonparticipant versus the intervention group.

As an observational study, we propose that findings were generalizable to a population of people with MSK pain with expressed interest in a digital MSK program. Similar to U.S. national estimates, our study included more females than males with chronic pain and more people who were overweight or obese than normal weight [45]. The study included people willing to participate in a digital health program and may not be generalizable to later adopters of health technology. We analyzed data collected from people who responded to surveys administered in app, by email, and by phone, and the results may not apply to non-respondents. However, we found that the baseline characteristics of the respondents was similar to that of the study sample (Table 1 and Additional File 1).

The study had the following limitations. First, this was an observational study and not a RCT. Thus, we cannot establish causality of the intervention’s effect on outcome improvement. In addition, the intervention group and nonparticipant group differed on baseline characteristics. But, we controlled for these measured variables in the adjusted models. Furthermore, results about improvement in depression and anxiety were among only those with moderate to severe symptoms at baseline.

Second, we may have omitted important confounding variables that attenuate outcomes estimates. For example, we did not collect data about medications that patients used before, during, or after the digital MSK program. Medication use may have influenced both program engagement as well as pain, function, and mental health outcomes. We cannot account for unmeasured factors like motivation. The intervention group may include people more motivated to manage pain and report pain improvement, thereby biasing our results upwards. We also did not collect medical diagnoses from study participants, and patients with different diagnoses may have pain with different attributes. As a result, we are unable to adjust for diagnosis as a confounding variable or use diagnosis in stratified analyses (e.g., analyze results for members with only inflammatory arthritis).

Third, our response rate for the nonparticipant group was lower than for the intervention group. The lower response rate in the nonparticipant group may have biased our estimates upwards if there were more nonrespondents in the nonparticipant group who also had improved outcomes. Finally, as a wholly digital health program, we include patient reported outcomes in this study and have not included any physician reported or objective assessments.

We propose the following research to build on the findings from this study. First, future studies can examine intermediate and long term follow-up for each care pathway (i.e., back, knee, shoulder, hip, neck) separately. Second, we recommend additional study of the interplay between pain and function and a digital MSK program. In the current study, improvements in pain at 12 month were not accompanied by the same magnitude of functional improvements. Third, we recommend studying the influence of engagement with a digital MSK program on clinical outcomes. Compared to those who engaged for only 3 months, people who engaged in the digital MSK program for 6 months were more likely to experience better pain outcomes in the long term. But engagement duration did not capture different aspects of engagement, including affective and cognitive investment, and how engagement may change over time [46].

## Conclusions

This study examined multiple clinical outcomes at three time points over a 12 month period. More participants of a digital MSK program experienced meaningful pain and functional improvement versus a nonparticipant group that never took part in the program. These results were demonstrated in the short, medium, and long term. We evaluated the program in real world settings so that results were more generalizable than results from tightly controlled clinical trials. We provided preliminary evidence about how a digital MSK program influenced use of traditional, in-person health care services. In conclusion, this study provided evidence that a digital MSK program may have had a lasting impact on improved pain, depression, and anxiety alongside decreased health care use.

## Supplementary Information

**Additional file 1.** Analytic sample characteristics.

**Additional file 2.** Descriptive results for pain and function, by subgroups defined by engagement duration.

## Data Availability

The datasets used and/or analysed during the current study are available from the corresponding author on reasonable request.

## Abbreviations

GAD-2: Generalized Anxiety Disorder 2-item scale
GAD-7: Generalized Anxiety Disorder 7-item scale
HOOS-PS: Hip Disability and Osteoarthritis Outcome Score Physical Function Short form
KOOS-PS: Knee Injury and Osteoarthritis Outcome Score Physical Function Short form
MCID: Minimally clinical important difference
MSK: Musculoskeletal
sf-NPAD: Neck Pain and Disability Scale short form
PHQ-2: Patient Health Questionnaire 2-item scale
PHQ-8: Patient Health Questionnaire 8-item scale
Q: Quarter
SPADI: Shoulder Pain and Disability Index
VAS: Visual analog scale
