# Supplementary material for: Clinical outcomes one year after a digital musculoskeletal (MSK) program: an observational, longitudinal study with nonparticipant comparison group
Source: BMC Musculoskelet Disord. 2022 Mar 11;23:237. doi: 10.1186/s12891-022-05188-x (PMC8914456; doi:10.1186/s12891-022-05188-x)
Supplement: Supplementary file 1 — Additional file 1. Analytic sample characteristics. [file 12891_2022_5188_MOESM1_ESM.docx]

# Additional File 1 Analytic sample characteristics

|  | **Comparison (N=447)** | **Intervention (N=2123)** |
| --- | --- | --- |
| **Gender** |  |  |
| Female | 262 (58.6%) | 1335 (62.9%) |
| Male | 181 (40.25) | 772 (36.4%) |
| Other | 0 (0.0%) | 5 (0.2%) |
| Prefer Not to Answer | 4 (0.9%) | 11 (0.5%) |
| **Age** |  |  |
| Mean (SD) | 46.2 (12.4) | 50.0 (11.9) |
| Median [Min, Max] | 47.0 [19.4, 87.5] | 51.1 [18.2, 86.2] |
| **BMI** |  |  |
| Underweight (<18.5) | 4 (0.9%) | 29 (1.4%) |
| Normal (18.5-24.9) | 92 (20.6%) | 611 (28.8%) |
| Overweight (25.0-29.9) | 148 (33.1%) | 686 (32.3%) |
| Obese (>30.0) | 203 (45.4%) | 797 (37.5%) |
| **Exercise Frequency** |  |  |
| Less than 1 hour | 179 (40.0%) | 535 (24.2%) |
| 1 to 2.5 hours | 167 (37.4%) | 919 (43.3%) |
| More than 2.5 hours | 101 (22.6%) | 669 (31.5%) |
