# Supplementary material for: Clinical outcomes one year after a digital musculoskeletal (MSK) program: an observational, longitudinal study with nonparticipant comparison group
Source: BMC Musculoskelet Disord. 2022 Mar 11;23:237. doi: 10.1186/s12891-022-05188-x (PMC8914456; doi:10.1186/s12891-022-05188-x)
Supplement: Supplementary file 2 — Additional file 2. Descriptive results for pain and function, by subgroups defined by engagement duration. [file 12891_2022_5188_MOESM2_ESM.docx]

# Additional File 2 Descriptive results for pain and function, by subgroups defined by engagement duration

| **Timepoint** | **Long Term** | **Long Term Total** | **Long Term %** | **Completer** | **Completer Total** | **Completer %** | **p-value** |
| --- | --- | --- | --- | --- | --- | --- | --- |
| 3 months | 810 | 1058 | 76.56% | 795 | 1065 | 74.65% | 0.165 |
| 6 months | 780 | 942 | 82.80% | 248 | 340 | 72.94% | <0.001 |
| 12 months | 308 | 404 | 76.24% | 202 | 302 | 66.89% | 0.004 |
